# The acute effects of a free acid beta-hydoxy-beta-methyl butyrate supplement on muscle damage following resistance training: a randomized, double-blind, placebo-controlled study

**DOI:** 10.1186/1550-2783-9-S1-P27

**Published:** 2012-11-19

**Authors:** Eric M Sikorski, Jacob M Wilson, Ryan P Lowery, NM Duncan, GS Davis, JA Rathmacher, SM Baier, MA Naimo, Stephanie MC Wilson, KA Dunsmore, J Walters, J Joy, TJ Morrison

**Affiliations:** 1 Department of Biology, The University of Tampa, Tampa, FL, USA; 2 Department of Health Sciences and Human Performance, The University of Tampa, Tampa, FL, USA; 3 Metabolic Technologies, Inc., Iowa State University Research Park, Ames, IA, USA; 4 Department of Animal Science, Iowa State University, Ames, IA, USA; 5 Department of Nutrition, IMG Performance Institute, IMG Academies, Bradenton, FL, USA

## Background

Beta-hydoxy-beta-methyl butyrate (HMB) when given over a two-week period of time (loading phase) has been demonstrated to decrease skeletal muscle damage, and improve recovery. However, few studies have investigated its acute effects on muscle damage and recovery. Therefore the purpose of this investigation was to determine the effects of short term free acid HMB (HMB-FA) supplementation on serum indices of muscle damage and perceived recovery following a high volume, muscle damaging training session.

## Methods

Twenty resistance trained males aged 21.3 ± 1.9 years with an average squat, bench press, and deadlift of 1.7± 0.2, 1.38 ± 1.9 and 2.07 ± 2.7 times their bodyweight were recruited for the study. Two weeks prior to and throughout the study subjects were placed on a diet consisting of 25 % protein, 50 % carbohydrates, and 25 % fat by a registered dietician who specialized in sport (RD, LDN, CISSN). All subjects participated in a high volume resistance training session consisting of 3 sets of full squats, bench press, deadlifts, pull-ups, bent over rows, shoulder press, barbell curls and triceps extensions. Prior to the exercise session subjects were randomly assigned to receive either a 3 g per day of HMB-FA (Combined with Food-grade orange flavors and sweeteners) or a placebo (Food-grade orange flavors and sweeteners) divided equally into servings given 30 minutes prior to exercise and with two separate meals on day 1. They were then instructed to consume the same amount of HMB-FA or placebo divided into breakfast, lunch and dinner on day two. Immediately prior to the exercise session and 48 hours post exercise, serum creatine kinase (CK), testosterone, cortisol, and perceived recovery scale (PRS) measurements were taken. Perceived Recovery Status consists of values between 0-10, with 0-2 being very poorly recovered with anticipated declines in performance, 4-6 being low to moderately recovered with expected similar performance, and 8-10 representing high perceived recovery with expected increases in performance. A 2 X 2 repeated measures ANOVA was used to assess any main effects. If any main effects were found LSD post hoc tests were incorporated to determine where those differences were located.

## Results

Significant time and group X time effects were found for CK, which increased to a greater extent in the placebo (140.7 ± 40.9 to 603.8 ± 249.0) than HMB-FA group (158.0 ± 50.9 to 322.2 ± 115.9) (p<0.05). There were also significant time and group X time effects for PRS, which decreased to a greater extent in the placebo (9.1 ± 1.2 to 4.6 ± 1.4) than the HMB-FA group (9.1 ± 0.9 to 6.3 ± 0.9) (p<0.05). There were no time or group X time effects for testosterone or cortisol.

## Conclusions

These results suggest that an HMB-FA supplement given over a short period of time (48 hours), and without a loading phase to resistance trained athletes can blunt increases in muscle damage and prevent declines in perceived readiness to train following a high volume, muscle damaging resistance training session.

